# Anatomical Variations of the Nasal Conchae and Nasal Septum and their Relationships with Alterations in the Maxillary Sinus Mucosa: A Study on Cone-beam Computed Tomography Images

**DOI:** 10.1055/s-0044-1788909

**Published:** 2025-01-27

**Authors:** Luara da Silveira Roberto Almeida, Aline da Silva Ruffo, Karina Lopes Devito

**Affiliations:** 1 Faculty of Dentistry, Universidade Federal de Juiz de Fora, Juiz de Fora, MG, Brazil; 2 Postgraduation Program in Dentistry, Faculty of Dentistry, Universidade Federal de Juiz de Fora, Juiz de Fora, MG, Brazil; 3 Clinical Dentistry Department, Faculty of Dentistry, Universidade Federal de Juiz de Fora, Juiz de Fora, MG, Brazil

**Keywords:** maxillary sinus, sinusitis, turbinates, nasal septum, cone-beam computed tomography

## Abstract

**Introduction** In the literature, there is divergence about the relationship between anatomical variations of the turbinates and nasal septum (NS) and alterations in the maxillary sinus (MS) mucosa.

**Objective** To determine, through cone-beam computed tomography (CBCT) images of Brazilian individuals, the prevalence and relationship of anatomical variations of the turbinates and NS with alterations in the mucosa of the MS, as well as to analyze the relationships of these variables with demographic data.

**Methods** The present cross-sectional study involved the analysis of 120 CBCT scans using the i-CAT Vision software, conducted by 2 calibrated examiners. The MS, lower and medium turbinates, and NS were evaluated. Data on gender, age, and the side affected by anatomical variation were also collected. The intra- and interexaminer agreements were assessed using Kappa indices. The association was analyzed using the Chi-squared or Fisher exact tests, and measured by the Phi, Cramer V, or Kendall Tau-C values.

**Results** Most patients presented partial opacification of the MS (89.2%), inferior turbinate hypertrophy (TH) (60.8%), and NS deviation (85%). There were no cases of inferior concha bullosa (CB), while the prevalence of middle CB was of 20%. Variation in the turbinates, CB, and NS were not significantly related to changes in the MS mucosa.

**Conclusion** We can conclude that, in the evaluated sample, there was no significant associations involving the studied variables.

## Introduction

Rhinosinusitis (RS) is an extremely prevalent disease that has a significant impact on the quality of life of affected individuals. 1 Its etiology may be related to physiological, pathological, or anatomical alterations, which prevent free drainage of the paranasal sinuses and cause secretion stagnation, increasing the risk of inflammation and infection. 2

A blockage in the ostiomeatal complex (OMC) results in impairment of sinus ventilation and mucociliary clearance, immune dysfunction, damage to epithelial defense, and biofilm formation. 2 Thus, one of the main causes of RS is the occlusion of the OMC due to anatomical variations, such as nasal septum (NS) deviation and changes in the nasal conchae. 1 2

Deviation of the NS can be described as a curvature of the septal contour to the right, to the left or shaped like an “S” within the nasal cavity. This anatomical variation can be congenital or due to trauma, 3 and it is a frequent alteration in the paranasal sinus region, increasing the risk of RS. 4 5 Although there is a study that reports a statistically significant correlation between NS deviation and RS, 1 other researchers have not observed such an association. 6

Pneumatized nasal conchae, also known as concha bullosa (CB), are a common anatomical variant of the paranasal sinus region. This change in the middle nasal conchae has been identified as a possible etiological factor for RS, 7 and an increase in the total volume of the nasal conchae has been associated with an increase in the thickness of the mucous lining of the maxillary sinus (MS). 8 However, controversial results have been found, 9 which support the hypothesis that CB and inferior turbinate hypertrophy (TH) do not affect the development of RS.

Furthermore, NS deviation and the location of nasal conchae alterations can affect each other. 10 11 Deviation of the NS interferes with the development of sinonasal structures, 12 and it is the most common incidental pathology that accompanies CB. 7 Although some authors 13 have observed an association between NS deviation and the presence of CB, the cause-effect relationship between them is still not very clear. For other authors, 6 no significant association between these structures was verified.

Regarding hypertrophy, there are few studies 13 on the association between NS deviation and inferior TH using cone-beam computed tomography (CBCT) images.

In the imaging evaluation of the paranasal region, including suspected nasal and paranasal inflammatory diseases, the tomographic exam is the gold standard; 3 14 CBCT consists of an imaging technique for dental use that, compared to conventional computed tomography, provides a lower dose of radiation, maintaining applications in the temporal bone, skull base and sinus image. 15

Knowledge of anatomy, variants, and the correct identification of sets of tomographic data help understanding the factors that favor RS, aiding in the diagnosis, patient counseling and planning of the most appropriate treatment for each case. 3 12 16

Although anatomical variations in this region are frequently found and play an important role in dysfunctional sinus drainage, it is still unclear in the literature whether they predispose to sinus pathology. The possible correlation regarding certain anatomical changes in the sinonasal region and pathologies of the paranasal sinuses is highlighted. Due to the contradictory results in the literature, 17 more research is needed to elucidate the effects of the anatomical variants of the sinonasal region.

Therefore, the present study aims to determine, through CBCT images of Brazilian individuals, the prevalence and the relationship of anatomical variations of the nasal conchae (CB and inferior TH) and of the NS (deviation) with mucosal alterations in the MS. Additionally, it aims to assess the relationship of these variables with demographic data (gender and age).

## Methods

The present cross-sectional observational study was carried out after approval by the Ethics Committee for Research with Human Beings of Universidade Federal de Juiz de Fora (UFJF; under opinion no. 1,942,848), and it included the analysis of 120 CBCT exams belonging to data from the Radiology Clinic the School of Dentistry of UFJF from November 2022 to April 2023.

The sample was selected using nonprobabilistic convenience sampling. Patients with exams that covered the MS, the lower and middle nasal conchae, and the NS were included. The exclusion criteria comprised history of facial trauma, fractures in the analyzed region, tumors, orthognathic or sinus surgeries, maxillofacial injuries, congenital craniofacial anomalies, and MS with the presence of biomaterial. In addition, patients under 18 years of age, as well as scans with acquisition errors, artifacts, and incomplete coverage of the area of interest were also excluded from the study. It is worth noting that the tomographic images used did not have any associated clinical data that would enable any inference about a specific patient diagnosis. The images were obtained for various dental reasons.

The CBCT images were acquired using the i-CAT Next Generation scanner (Imaging Sciences International, Inc., Hatfield, PA, United States), with the acquisition protocol of 5 mA, 120 kV, 0.25 mm voxel, minimum field of vision (FOV) of 7 × 23 cm, and 40 s of rotation time.

The exams included in the present study were evaluated using the i-CAT Vision software (Imaging Sciences International, Inc.), which provided images in axial, sagittal, and coronal sections with a slice thickness of 0.25 mm. The exams were evaluated independently by two trained and calibrated examiners, who used the same computer, in a silent environment with good lighting.

Thus, initially, each examiner randomly analyzed 20 exams that were not part of the final sample, which were reassessed after a 1-week interval to verify the calibration. Only after reaching intraexaminer agreement rates greater than 70% did the evaluators proceed to analyze the study sample. In the final sample, in case of disagreements regarding the evaluation of the tests, the definitive diagnosis would be established by consensus between the examiners.

The identification of the presence or absence of anatomical variations was performed by evaluating the CBCT coronal sections, on a multiplanar reconstruction (MPR) screen. The images were evaluated bilaterally, and data were collected regarding gender, age group, and side affected by the alteration in anatomy.

**Fig. 1:**
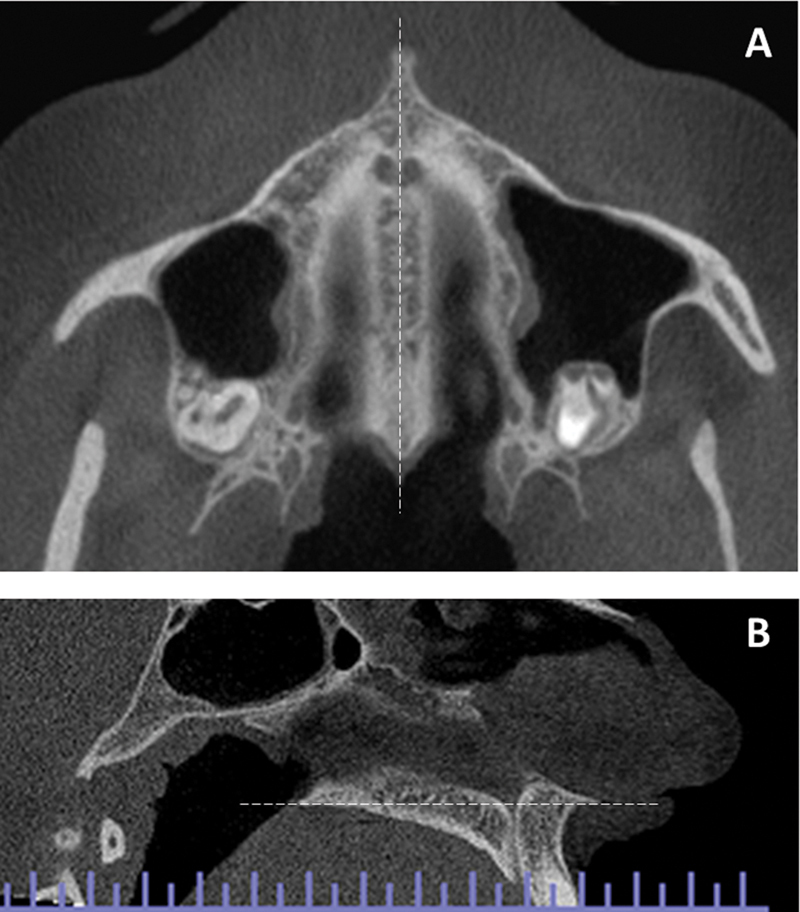
Adjustment of the palate and nasal spines for analysis. ( **A** ) Axial section. ( **B** ) Sagittal section.

The potential for asymmetries due to incorrect patient positioning and the absence of standardized positioning were considered. Reference lines were used to adjust the measurement plane for all patients. Thus, before the analyses, the nasal spines in the exams were adjusted using sagittal and axial cuts, aligning a central line between the anterior and posterior nasal spine ( Fig. 1 ). Furthermore, software tools were used to adjust the brightness, contrast, and zoom of the images, enhancing visualization.

The structures evaluated were the MS, the inferior and middle nasal conchae, and the NS. The classification was divided into absence of anatomical variation (0) or presence of anatomical variation (1); the variations evaluated in the present study were the inferior CB, middle CB and inferior TH.

Concha bullosa consists of the pneumatization of a nasal concha or the presence of an air cell in it. 3 The presence of CB was determined based on the criteria established by Stallman et al. (2004). 19 Thus, it was considered present when more than 50% of the vertical height (measured from superior to inferior) of the nasal conchae was pneumatized in the coronal section. 13 18 19

The criterion to determine the inferior TH was based on the maximum width of the lower nasal conchae in the CBCT coronal section. Thus, values greater than 10 mm were considered inferior TH. 13 20

**Fig. 2:**
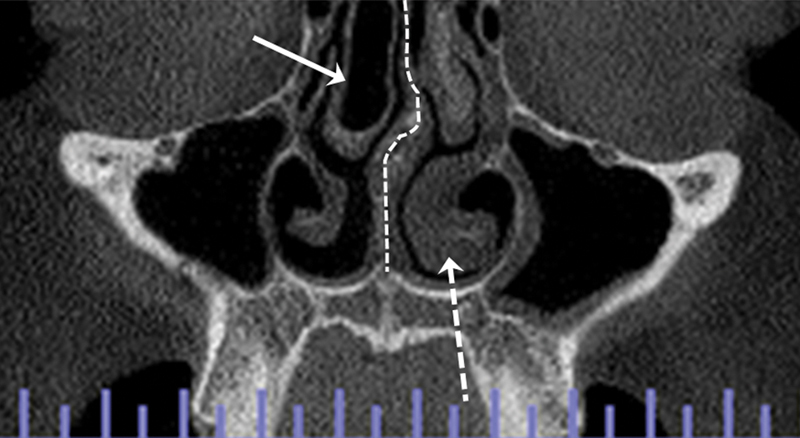
Coronal section. Middle concha bullosa (CB) (right side), inferior turbinate hypertrophy (TH) (left side), and nasal septum (NS) deviation.

The NS deviation was categorized as absent (0), to the right side (1), to the left side (2), or in “S curvature” (3). Deviation of the NS consists of any deviation of the septal contour towards one side of the nasal cavity or in the shape of an “S”; 3 it was determined on coronal images, and the direction of the deviation was attributed to the convex side. 4 6 Figure 2 shows a case of middle CB, inferior TH, and NS deviation.

**Fig. 3:**
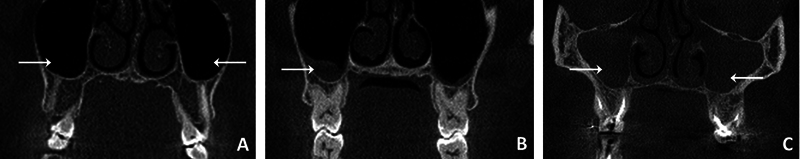
Coronal sections illustrating mucosal changes in the maxillary sinus (MS), classified according to the Lund Mackay system. ( **A** ) Score of 0 (no opacification). ( **B** ) Score of 1 (partial opacification). ( **C** ) Score of 3 (complete opacification).

Mucosal alterations in the MS were analyzed and classified according to the Lund Mackay system, which is widely used for the radiological staging of RS. 15 It is based on the score of each sinus, which ranges from 0 to 2 points: 0–no opacification; 1–partial opacification; and 2–complete opacification ( Fig. 3 ). 1 14 15 16

### Statistical Analysis

To assess the intra- and interexaminer agreement, Kappa indices were calculated, considering the following interpretation for analysis: 0–no agreement; 0.01 to 0.20–poor agreement; 0.21 to 0.40–fair agreement; 0.41 to 0.60–moderate agreement; 0.61 to 0.80–strong agreement; 0.81 to 0.99–almost perfect agreement; and 1–perfect agreement. 21

To verify the associations involving the qualitative variables, the Chi-square or Fisher exact tests were used. The strength of the association was determined by the Phi or Cramer V values for the nominal variables, and by the Kendall Tau-C value for the ordinal variables. The statistical analyses were conducted using the IBM SPSS Statistics for Windows (IBM Corp., Armonk, NY United States) software, version 21.0, with a significance level of 5% ( *p* ≤ 0.05).

## Results

The tomographic images of 120 (55% female and 45% male) patients aged between 18 and 76 (mean ± standard deviation [SD]: 38.82 ± 17.13) years were evaluated bilaterally.

The results of the agreement between the examiners were all significant ( *p* ≤ 0.05), with Kappa indexes ranging from 0.59 to 1 (mean ± SD: 0.64 ± 0.22) for the interexaminer agreement, and from 0 .79 to 1 (mean ± SD: 0.89 ± 0.10) for the intraexaminer agreement, which were considered strong and almost perfect agreements respectively. 21

Table 1 illustrates the descriptive data of the sample in relation to the studied variables. Most patients presented partial opacification of the MS (89.2%), inferior TH (60.8%) and NS deviation (85%). There were no cases of inferior CB, while the prevalence of middle CB was of 20%.

**Table 1:** Descriptive data of the study sample in relation to the studied variables

	n	%
**Maxillary sinus** mucosal alteration*		
Absent	15	6.3
Partial opacification	214	89.2
Total opacification	11	4.6
Inferior concha bullosa*		
Absent	240	100
Present	0	0
Middle concha bullosa*		
Absent	192	80
Present	48	20
Inferior turbinate hypertrophy*		
Absent	94	39.2
Present	146	60.8
**Nasal septum deviation**		
Absent	18	15
To the right side	44	36.7
To the left side	45	37.5
“S”-shaped	13	10.8

**Note:** *Bilateral assessment.

Table 2 shows that there were no significant associations regarding the studied variables. Variation in the turbinates, CB, and NS were not significantly related to changes in the MS mucosa.

**Table 2:** Associations of the different variables studied with maxillary sinus mucosa alterations

	Maxillary sinus mucosal alteration			Total	*p* -value*
	0	1	2		
**Middle concha bullosa**	3 (6.25%)	43 (89.58%)	2 (4.17%)	48	0.921
**Inferior turbinate hypertrophy**	8 (5.48%)	131 (89.72%)	7 (4.80%)	146	0.568
Nasal septum deviation**	6 (5.88%)	91 (89.22%)	5 (4.90%)	102	0.306

**Notes:** *Chi-squared or Fisher exact test. **Only the presence or absence of nasal septum deviation was considered.

## Discussion

The relationship between anatomical variations in the nasal and paranasal regions and the thickening of the MS mucosa is an essential field of study, with direct implications for the diagnosis, treatment, and management of various clinical conditions. Understanding these relationships is crucial to improve patient outcomes and advance the medical practice. Accurate diagnoses of these variations yield many advantages, such as aiding in the identification of underlying causes of patient symptoms, minimizing risks in endoscopic surgeries, enabling the prescription of personalized treatments based on each variation, preventing complications by implementing preventive measures in patients with anatomical variations that hinder drainage, enabling more effective management of conditions such as nasal congestion, facial pain, and headaches, thereby alleviating symptoms and improving patients' quality of life.

Pneumatized nasal conchae, also known as CB, is a common anatomical variant in the region of the paranasal sinuses. 7 22 Different prevalence rates have been reported in the literature, such as 46, 23 37.7, 6 31.7, 7 28.37, 10 and 28.06%, 22 which are comparable to the 20% found in the present study.

Regarding CB and gender, the literature reports 6 a prevalence of 38.8% in male subjects and 37% in female patients, with no statistically significant difference. This lack of significant association between CB and gender is consistent with the findings of the present study.

Inferior CB, on the other hand, is a rare abnormality in the paranasal sinuses, 24 25 and no case was found in the sample of the present study. Although a previous study 24 associated this anatomical variation with sinonasal symptoms, including nasal obstruction, headache, and NS deviation, none were observed in the current study.

When the studied variable was correlated with age, no significant association was found, which is consistent with the literature. 25 Similarly, there was no significant correlation between inferior CB and gender. The literature reports 25 similar frequencies among men and women, of 1.57 and 1.88% respectively, with no significant association. However, Alnatheer and Alkholaiwi 24 observed higher prevalences, of 30.8% in males and of 69.2% in females.

The anatomical variations most associated with inferior CB were CB in the other nasal conchae and NS deviation. Additionally, it is difficult to clinically differentiate this alteration from inferior TH, with the diagnosis established through computed tomography. Therefore, it is important to consider this condition, especially in cases unresponsive to medical treatments, and to opt for surgical intervention when symptoms persist. 25

Regarding the inferior TH, previous studies 8 have shown a prevalence of 65 in males and 35% in females. However, no statistically significant association was found between gender or age and the total volume of nasal conchae, 8 20 which is in line with the results of the present study.

Another anatomical alteration discussed is NS deviation. The NS consists of a crucial support structure for the nasal cavity that can present several anatomical variations, 26 including deviation. 2 26 This common malformation increases the likelihood of nasal obstruction, RS, upper-airway and middle-ear infections, and it can impact activity, esthetics, breathing, and the vocal cords. 5 However, NS deviation can also be mild and asymptomatic. 3

Septal deformities are much more common than a nondeviated septum, 3 with NS deviation found in 85% of patients. This finding is consistent with that of other studies in the literature, which report prevalence rates ranging from 56 to 92.7%. 4 5 6 9 10 23 26 27 28 The mean age of the patients with NS deviation (43.95 years) was significantly higher than that of patients without it (33.37 years), indicating that the incidence of this anatomical variation increases with age. 5 However, Shetty et al. 13 did not observe significant differences regarding the different types of NS deviation and age group, nor with NS deviation in general.

In the present study, no significant association was found between NS deviation and gender or age. However, other studies have shown a male predominance, 26 29 with rates of 67.4 among female patients and of 80% among male subjects, indicating a statistically significant difference. 26 Despite this, some studies 5 6 13 28 also report a male predominance without a statistically significant relationship in the frequencies of NS deviation between genders.

Deviation of the NS and anatomical variations in the nasal conchae can influence each other. 10 The width of the inferior TH and the presence of CB can significantly impact the degree of NS deviation. 13 Additionally, the total volume of the nasal conchae tends to increase with the degree of NS deviation. 8 However, other authors 12 have found that the incidence of CB is not affected by the degree of NS deviation.

According to Kalaiarasi et al., 7 the most common incidental pathology accompanying CB was NS deviation. In the study by Al-Rawi et al., ^6^ although the relative frequency of CB was higher among individuals with NS deviation (40.5%) compared to those without it (29.6%), the association was not statistically significant. In the present study, no significant correlation was observed involving variations in the anatomy of the nasal conchae and NS deviation. On the other hand, Lee et al. 10 observed a significant relationship between the orientation of NS deviation and CB, with CB mainly located in the concave cavity of the NS deviation. Additionally, both CB and inferior TH were more prevalent on the side contralateral to the unilateral NS deviation, 9 with the mucosal thickness and diameter of the lower ipsilateral nasal conchae significantly greater than those on the contralateral side. 30

Liu et al. ^11^ reported that lower nasal conchae widths on the concave side were greater than those on the convex side due to compensatory hypertrophy of the lower nasal conchae mucosa on the concave side and atrophy of the nasal conchae bone caused by NS deviation on the convex side. Furthermore, there was a positive and significant correlation between the degree of NS deviation and the total volume of the lower nasal conchae, which was greater in patients with NS deviation. This suggests that, in the presence of NS deviation, there is a significant increase in the total NS volume in patients with unilateral NS hypertrophy. 20 However, in the sample of the present study, no significant correlation was found between inferior TH and NS deviation.

Although NS deviation and CB location can affect each other, Lee et al. 10 found no significant relationship between MS and either NS deviation or CB, 10 which aligns with the results of the current study.

### Evaluation of the Maxillary Sinus Mucosa

The anatomy of the nasal cavities and paranasal sinuses is one of the most diverse in the human body, 31 with frequent anatomical variations that significantly impact sinus drainage. However, the literature is unclear on whether these variations predispose individuals to sinus pathology. 17 Furthermore, these anatomical differences vary among ethnic groups. 31 Therefore, it is important to evaluate the prevalence and associations of these alterations within the Brazilian population.

When the studied variables were correlated with age, no significant association was found in the present study. However, the literature 29 suggests that RS mainly affects young adults. Nevertheless, Alsaggaf et al. 4 reported that older individuals present a higher risk of developing RS. In addition, Bekin Sarikaya and Bayar Muluk 30 found that ipsilateral RS was present in 34% of individuals under and in 52% of those over 35-years-old.

Regarding gender, the present study did not find significant associations. However, some studies 4 have reported significant associations between RS and gender, with men being about twice as likely to develop RS compared to women. In contrast, Al-Rawi et al. 6 observed a significantly higher prevalence of sinus pathology in female subjects than in male patients.

The MS is the most affected, 7 with the most frequent anatomical variations including NS deviation and CB. 3 31 These variations are also among the primary anatomical alterations that lead to OMC obstruction, 1 2 which is a major cause of RS, and its blockage results in impairment in sinus ventilation and mucociliary clearance, immune dysfunction, compromised epithelial defense, and biofilm formation. 2

Deviation of the NS has been linked to the pathogenesis, progression, and severity of RS, with mechanical and aerodynamic theories explaining this relationship in ipsilateral cases. 2 Anatomical variants of the NS may be associated with a higher incidence of RS due to structural and functional alterations, 26 as NS deviation interferes with sinonasal development by affecting pneumatization. 12 29 Thus, NS deviation is one of the most common causes of OMC obstruction, which can interfere with adequate airflow and potentially predispose individuals to RS. 1 However, as in the present research, other studies 6 10 did not show a significant association between NS deviation and RS.

Regarding the presence of CB, as observed in the current study, other findings in the literature 6 7 9 10 22 indicate that this variation is not significantly associated with sinus pathology. Therefore, anatomical variations can interfere with the sinus mucociliary drainage pathway, leading to infection, but correlations between anatomical variations and RS symptoms are not always evident. 23 Furthermore, NS deviation and CB were common radiographic findings in asymptomatic patients in the study by Al-Rawi et al.. 6

The relationship between the volume of the lower nasal conchae and the thickness of the MS mucous lining is an important parameter in the assessment of sinonasal diseases. 8 A regression analysis conducte3d by Shetty et al. 8 revealed that increasing the thickness of the sinus lining correlated significantly with an increase in total nasal conchae volume, suggesting that individuals with radiographically evident MS lining had greater total nasal conchae volume. However, the present research, like others, 9 found no evidence that inferior TH affects the development of RS.

In the current study, total MS opacification was observed in 4.6% of the patients, while partial opacification, in 89.2%. The literature reports varying prevalence rates for RS, ranging from 24% to 51.2%. 4 10 27 It is important to note that thickening of the sinus mucosa is merely a radiographic sign of RS; clinical data are essential for an accurate diagnosis. In the present study, the tomographic images were obtained from an image bank and lacked associated clinical data. Therefore, it was not possible to associate the images with any pre-established clinical diagnosis of RS.

Understanding anatomical variations and their association with the incidence of RS can offer valuable radiological insights that aid in patient management. 26 However, the diagnosis of RS cannot be based only on imaging tests, as a considerable number of asymptomatic patients exhibit anatomical abnormalities on CT or magnetic resonance imaging scans. Therefore, the validation of diagnostic accuracy depends on the reliability of the clinical history and related tests, such as endoscopy and histopathology. 14 15 Consequently, RS should be initially evaluated through a detailed anamnesis, followed by complementary exams if necessary. 1

## Conclusion

Deviation of the NS and inferior TH are common anatomical variations in the Brazilian population studied, while CB is less frequent. However, the anatomical variations studied were not significantly associated with alterations in the MS mucosa.
